# Items

**Published:** 1907-11

**Authors:** 


### ITEMS.

---

MESSRS. Battle & Company, of Saint Louis, have recently issued pamphlet No. 3, of the series of dislocations. The upward dislocation of the sternal end of the clavicle is contained in this number.

---

THE State Civil Service Commission will hold examinations November 16, 1907, for, among others, the following positions: Dentist, State Institutions; Health Officer, Village of Port Chester, $600; Librarian for the Blind, State Library, $720; Physician, State Hospitals, $900 and maintenance; Trained Nurse, State Institutions, $420 to $600, and maintenance; Typewriter Copyist, State and County Offices.

The last day for filing applications for these positions is November 9. Full information with application forms for any of these examinations may be obtained upon postal card request from the Chief Examiner of the Commission, Charles S. Fowler, Albany.

